# Fellowships, Grants, & Awards

**Published:** 2004-08

## NHLBI Clinical Proteomics Programs

This Request for Application (RFA) will establish Clinical Proteomics Programs to promote systematic, comprehensive, large-scale validation of existing and new candidate protein markers that are appropriate for routine use in the diagnosis and management of heart, lung, blood, and sleep diseases. These programs will facilitate validation of protein panels that may be used to predict disease susceptibility or to assist in differential diagnosis, disease staging, selection of individualized therapies, or monitoring of treatment responses. In addition, this RFA seeks to establish a high quality education and skills development program to encourage and ensure that scientists develop competencies and expertise needed to address the complex, multifaceted challenges in clinical proteomics.

Heart, lung, blood, and sleep diseases are major causes of morbidity and mortality. Cardiovascular disease is the number one killer in the United States in both men and women, across all major racial groups and totals nearly one million deaths a year. Lung diseases such as chronic bronchitis, emphysema, asthma and other obstructive or interstitial conditions account for more than 230,000 deaths annually, placing an enormous burden on our healthcare system. Blood diseases such as venous thrombosis and pulmonary embolisms are causes of significant public health concern, as well. Sleep disorders and insufficient sleep represent severe health concerns for tens of millions of Americans.

Improving patient care through the use of protein markers is well established clinically. For example, the definition of heart attack, as well as the determination of the benefit derived from antithrombotic treatments, rests on serum troponin measurement. The detection of extremely small quantities of this protein identifies patients at high risk for adverse outcomes as well those that will derive greater benefit from antithrombotic and other interventional strategies. Assay of the B-type natriuretic peptide also contributes to standard clinical information in the diagnosis of congestive heart failure. Myeloperoxidase was recently shown to help in the diagnosis of atherosclerosis and acute coronary syndromes.

The predictive values, sensitivity, and specificity of many of the individual protein markers, currently in clinical use, could potentially be enhanced if analyzed and measured in a panel. Observational studies have shown that combining protein markers troponin I, C-reactive protein and B-type natriuretic peptide into panels can provide valuable information on stratifying risk for acute coronary syndromes. Panels of protein markers, appropriately validated, could facilitate better and earlier diagnosis, improve disease staging and selection of individual therapies and lead to more reliable monitoring of treatment responses, leading to substantial improvements in public health.

The application of proteomics in the clinical environment is limited due to a lack of knowledge regarding which proteins are most useful for analysis and how data are interpreted and represented. Important research needs include the identification of panels of protein markers that are likely to provide useful clinical information, design of practical assays for these panels, and validation of these panels and assays in well characterized populations of human subjects. The emergence of clinical proteomics promises major advances in disease management, provided that a continuous channel exists for translating protein discoveries into tangible clinical benefits.

The purpose of this RFA is to establish an infrastructure for research teams to validate protein panels and to measure multiple candidate markers accurately, for heart, lung, blood, and sleep diseases. The Clinical Proteomics Programs established for this purpose will design panels of candidate proteins for disease areas, develop high throughput analytic methods, assess the predictive value of these proteomic measurements using biological specimens and clinical data from existing study populations, and establish procedures and standards for quality control.

A major shortfall of clinical proteomics is the lack of a robust infrastructure for clinical candidate panel validation. Validation is necessary to confirm the relationship to the target disease in large numbers of patient samples and requires highly standardized protein measurement systems. The samples must be derived from well characterized sample sets with associated high-quality clinical information. The validation process provides the critical evidence necessary for translating protein knowledge into practices impacting public health. A significant opportunity now exists to enhance the validation stage and help translate protein discoveries into clinical practice. Many completed and ongoing clinical trials and epidemiologic studies have disease associated biological samples in addition to detailed clinical data. Leveraging this investment will enhance validation efforts.

Panels of protein markers in the following areas would represent appropriate topics for proposed projects. This list is not intended to be all-inclusive, and other topics should be considered. 1) Predict susceptibility to coronary artery disease or acute and chronic pulmonary disease; 2) Assess the severity and rate of progression of atherosclerosis or pulmonary disease; 3) Differential diagnosis for patients presenting with shortness of breath, chest pain or elevated blood pressure; 4) Detect occult myocardial infarction and subclinical cardiac disease and/or damage; 5) Select optimal, individualized medical management strategies; 6) Monitor therapeutic and adverse responses to antihypertensive drugs or drugs for asthma and other lung diseases such as inhaled corticosteroids, bronchodilators, and leukotrienes; 7) Identify early stages of pulmonary disease before significant pathogenesis has occurred; 8) Evaluate risk of thrombosis in individuals with a predisposition to cardiovascular disease or stroke; 9) Evaluate risk of bleeding and appropriate management strategies in patients with bleeding disorders - hemophilia, autoimmune blood disorders, von Willebrand disease; 10) Manage anticoagulation therapy in patients with thromboembolic disorders; 11) Identify markers for early diagnosis and prognosis of heart, lung, blood, and sleep disorders; 12) Develop tests to rapidly and accurately distinguish thromboembolic stroke from hemorrhagic stroke.

Projects outside the scope of this RFA will not be considered responsive and include: 1) Studies that do not address heart, lung, blood, or sleep disorders; 2) Studies that are focused on developing new proteomic technologies to identify protein markers 3) Proteomic discovery efforts.

We encourage inquiries concerning this RFA and welcome the opportunity to answer questions from potential applicants. It is highly recommended that prospective applicants contact program staff (please see the “Contact” section below) about proposed projects.

A Clinical Proteomics Program should be an identifiable organizational unit formed by a single institution or a consortium of cooperating institutions. Each Clinical Proteomics Program must provide a multidisciplinary team structure, ensuring effective coordination and integration between the selection and validation components of the Program. The team should encompass multi-disciplinary expertise and should include proteomic researchers, bio-engineers, clinical chemists, protein chemists, experts in biostatistics and bioinformatics, clinical investigators, and epidemiologists.

The marker selection process should focus on the design of protein marker panels that are most useful in clinical situations with under met needs. The team should primarily be responsible for prioritizing candidate protein markers and panels for validation. The selection component should actively develop candidate protein marker panels from a wide range of sources, such as proteomic discovery efforts, published reports, differential expression based research studies and in silico sequence-based predictions.

The use of biological samples obtained by minimally invasive methods (e.g., blood, sputum, and urine) is encouraged. Samples from ongoing studies can also be used provided appropriate Institutional Review Board (IRB) amendments to existing protocols have been obtained.

Since quantitative measurements of candidate markers in large and well defined clinical samples is central to the validation effort, criteria for the selection of the source material as well as the criteria for validation of the candidate markers must be specified. Where possible, existing technology platforms should be explored as multiplexing tools during panel development. Efforts to minimize sample consumption are encouraged to ensure the maximum number of assays. Emphasis will be placed on development of panels with high predictive value, specificity, and sensitivity; development of flexible assay protocols to accommodate the inclusion of newly identified proteins into ongoing validation efforts; refinement and development of innovative biostatistical tools and methods for selection of protein marker panels and for increasing the diagnostic sensitivity and specificity; and assay development applicable to clinical settings.

The multidisciplinary team will also evaluate pre-analytic issues, (including those relating to sample collection, storage, processing, and handling), and set criteria, standardize, and implement preanalytic protocols prior to validation. Each Clinical Proteomics Program should have access to characterized samples with well defined clinical data and the appropriate IRB approvals before funding. Furthermore, they should operate on an ‘open source’ model system, making the data, statistical and bioinformatic tools that are generated and developed in the programs, accessible to the public domain within a time period to be determined at the first meeting of the Inter-Program Steering Committee.

An Inter-Program Steering Committee (with membership from all the programs) will be appointed and will have scientific management oversight and responsibility for developing communication, coordination and collaboration among the Programs. In addition, there will be an External Scientific Panel, advisory to the National Heart, Lung and Blood Institute (NHLBI) that will evaluate the progress of the Clinical Proteomics Programs.

In order to facilitate the functions that are common to each program, one of the programs will be selected to function as an Administrative Coordinating Center (ACC) for all the programs. Therefore, applicants must include as a separate section in their proposal, a description of an Administrative Coordinating Center that will be reviewed separately, independent of the scientific application. Specification for the ACC application can be found under the section, “Packaging the Clinical Proteomics Program Application”.

Each program is expected to develop mechanisms towards education of skills necessary for clinical proteomics. Full implementation of a nationwide effort in translational research for clinical proteomics requires availability of trained M.D., M.D. /Ph.D., and Ph.D. scientists. These individuals must be knowledgeable about the diverse aspects of clinical proteomics and able to integrate the translational and clinical concepts necessary for application to heart, lung, blood, and sleep diseases. One unique feature of the Clinical Proteomics Program is to function as a spring board for advancing education, at the National level, by establishing various mechanisms, such as specialized short courses, and ‘hands on’ programs that will focus on guiding graduate students, trainees, technical personnel, M.D./Ph.D. and Ph.D. scientists in translation research for clinical proteomics. Both Clinical Proteomics Program and NHLBI-supported investigators would be eligible for these educational opportunities.

This RFA will use the National Institutes of Health (NIH) cooperative agreement (U01) award mechanism. In the cooperative agreement mechanism, the Principal Investigator retains the primary responsibility and dominant role for planning, directing, and executing the proposed project, with NIH staff being substantially involved as a partner with the Principal Investigator, as described under the section "Cooperative Agreement Terms and Conditions of Award"

Prospective applicants are asked to submit a letter of intent that includes the following information: descriptive title of the proposed research; name, address, and telephone number of the Principal Investigator; names of other key personnel; participating institutions; number and title of this RFA. Although a letter of intent is not required, is not binding, and does not enter into the review of a subsequent application, the information that it contains allows NHLBI staff to estimate the potential review workload and plan the review.

Applications must be prepared using the PHS 398 research grant application instructions and forms (rev. 5/2001). Applications must have a Dun and Bradstreet (D&B) Data Universal Numbering System (DUNS) number as the Universal Identifier when applying for federal grants or cooperative agreements. The DUNS number can be obtained by calling 866-705-5711 or through the web site at http://www.dunandbradstreet.com/. The DUNS number should be entered on line 11 of the face page of the PHS 398 form. The PHS 398 is available at http://grants.nih.gov/grants/funding/phs398/phs398.html in an interactive format. For further assistance contact GrantsInfo, 301-435-0714, email: GrantsInfo@nih.gov.

Each application to establish a Clinical Proteomics Program must be submitted as one application by a Clinical Proteomics Program Director, who will be responsible for organizing and maintaining effective integration and interaction of the program. A clear description of interaction among the various components, plans for communication, collaboration and sharing among investigators in the Clinical Proteomics Program should be included. The Clinical Proteomics Program Director should also indicate the mechanism for handling day-to-day administrative details, program, coordination, planning and evaluation. The director will be required to have a minimum of 25 percent level of effort, and the responsibility of oversight and coordination of all projects or components of the Program, whether or not they are at his/her institution. Each program should clearly outline its administrative and organizational structure.

Applications should include appropriate budget forms providing adequate budget justification with all applicable direct and facilities and administrative costs. Estimating of staffing needs, including principal investigator, other professional and support staff must be included. During the course of the project period, it is anticipated that technologies will improve and the proposed studies may change. Accordingly, it is expected that the principal investigators will be allowed adjustments in their scientific projects to accommodate such things. Budgets should include travel costs for Awardees Meetings and Inter-Program Steering Committee Meetings, as detailed under the section titled, “Special Requirements” along with statements indicating willingness to participate in these meetings and abide by its governance.

An educational component is another integral part of each Clinical Proteomics Program. A clear description of the efforts to educate and cross train across disciplines of clinical proteomics must be outlined, including the plans for developing short courses and ‘hands on’ programs. The process of selection and monitoring of candidates for these educational activities must be portrayed as well.

A separate section not exceeding 5 pages, detailing plans for an Administrative Coordination Center, should be included in each Clinical Proteomics Program application. This section should be placed following the section on the Research Plan. The Center will facilitate functions common to all the Clinical Proteomics Programs, coordinate meetings of the awardees, the Inter-Program Steering Committee and the External Advisory Panel, and manage a Clinical Proteomics Program intranet website. The Center will also be responsible for setting up the monthly conference calls of the Steering Committee. This section should also include separate budget justification pages for the operation of the Administrative Coordination Center not to exceed 100,000 direct costs in any year. Applications should provide adequate budget justification with all applicable direct and facilities and administrative costs, including estimated costs associated with the travel of the External Advisory Panel (6–8 members). Estimation of staffing needs and communication costs must be included. The award will be subject to administrative review annually.

Applications not conforming to these guidelines will be considered unresponsive to this RFA and will be returned without further review.

The RFA label available in the PHS 398 (rev. 5/2001) application form must be affixed to the bottom of the face page of the application. Type the RFA number on the label. Failure to use this label could result in delayed processing of the application such that it may not reach the review committee in time for review. In addition, the RFA title and number must be typed on line 2 of the face page of the application form and the YES box must be marked. The RFA label is also available at: http://grants.nih.gov/grants/funding/phs398/labels.pdf.

The Center for Scientific Review (CSR) will not accept any application in response to this RFA that is essentially the same as one currently pending initial review, unless the applicant withdraws the pending application. However, when a previously unfunded application, originally submitted as an investigator-initiated application, is to be submitted in response to an RFA, it is to be prepared as a new application. That is, the application for the RFA must not include an Introduction describing the changes and improvements made, and the text must not be marked to indicate the changes from the previous unfunded version of the application.

Letters of intent are due 17 September 2004. Applications are due 14 October 2004. The earliest anticipated start date is July 2005.

Contact: Pothur R Srinivas, Division of Heart and Vascular Diseases, NHLBI, 6701 Rockledge Drive, Rm 10188, Bethesda, MD 20892-0001 USA, 301-435-0550, fax: 301-480-2858, email: ps241q@nih.gov; Anne P. Clark, Chief, Review Branch, Division of Extramural Affairs, NHLBI, 6701 Rockledge Drive, Rm 7214, MSC 7924, Bethesda, MD 20892-7924 USA, Bethesda, MD 20817 (for express/courier service), 301-435-0270, fax: 301-480-0730, e-mail: ac42y@nih.gov.

Reference: RFA No. RFA-HL-04-019

## New Technology for Proteomics and Glycomics (SBIR/STTR)

Notice: this program announcement (PA) must be read in conjunction with the current Omnibus Solicitation of the National Institutes of Health (NIH), Centers for Disease Control and Prevention (CDC), and Food and Drug Administration (FDA) for Small Business Innovation Research (SBIR) and Small Business Technology Transfer (STTR) Grant Applications. The solicitation (see http://grants.nih.gov/grants/funding/sbirsttr1/index.pdf or http://grants.nih.gov/grants/funding/sbirsttr1/index.doc) contains information about the SBIR and STTR programs, regulations governing the programs, and instructional information for submission. All of the instructions within the current SBIR/STTR Omnibus Solicitation apply.

The principal limitations in the field of proteomics are technological in nature. Proteomics, and the sub-discipline of glycomics, are rapidly developing, technology-intensive fields. Separations, mass spectrometry, microarray, bioinformatics, and other tools have advanced rapidly to support the explosive growth of biomedical applications in this area. However, technologies and methods remain largely inadequate to address the majority of meaningful biological problems, particularly with respect to quantitative and real time measurements. Continued intensive development of advanced tools is essential to meet two needs. First, improvements in basic bioanalytical technologies are essential to these endeavors. This includes but is not restricted to robotics, sample preparation and pre-fractionation, analytical separations, gel and array imaging, quantitation, mass spectrometry, intelligent automated data acquisition, and database searching. Second, improved informatics technologies are essential for the conversion of data into meaningful results and interaction models. Improved informatics tools will also facilitate the integration and synergistic development of the basic analytical tools mentioned above. Additionally, the translation of advances in proteomics to a clinical setting should be a priority.

Proteomics is a rapidly expanding field. Many of the potential scientific and medical rewards of proteomics’ successful application to complex systems seem deceptively near. A broad range of technologies is evolving rapidly to meet the needs of the field. However, despite explosive growth in both academic and commercial efforts, concrete technical capabilities are far from adequate to realize this promise. Proteomics technologies and methods in the three broad, interacting domains of biology, analytical chemistry, and informatics are still largely inadequate to address the bulk of challenging biological problems. This is the case with respect to both core capabilities and scale.

The broad scope of proteomics might perhaps be broken down into six types of questions that are addressed in some form: (1) identification of individual proteins, (2) recognition of protein interactions, (3) relative quantitation to distinguish differential expression of proteins, (4) characterization of post-translational modifications, (5) qualitative or quantitative measurements at high spatial and/or temporal resolution to address the dynamics of protein interactions, and (6) formulation of models based on results from components 1–5.

The categories above define the type of information being sought, and imply the need for technologies capable of addressing the challenges inherent in each type of experiment. Those specific technologies may reside within any of the three domains that define proteomics, or may function as a bridge between them. For example, tools for tissue or subcellular fractionation may reside squarely in the biological domain, but could also be designed in such a way as to maximize synergy with widely used analytical separations methods.

It is important that in a field as complex and interdisciplinary as proteomics, technology development be pursued with a sound understanding of context. One area of particular interest is the development of technologies that will permit observations to be quantitative and made in real time, whether for clinical studies or experimental systems.

In addition to the development of broadly applicable research tools that address the core technical challenges in proteomics, unique constraints in two subordinate areas merit special attention. We especially encourage applications in response to this announcement that address the unique needs of glycomics and clinical proteomics, described below.

The application of proteomics tools in the clinical setting lags far behind their use in basic science and drug discovery. Though this is not due solely to technological constraints, the unique challenges associated with development of simple, rapid, and robust technologies for the clinic demand a somewhat different perspective than might be taken in consideration of a purely research-driven project. Likewise, this difference in perspective and priorities should open the possibility of approaches that might be wholly inadequate from a research perspective but may be appropriate in the clinic. Finally, the exploitation of insights previously developed in research-oriented proteomics to develop more specific, robust tools for clinical applications is also an appropriate goal.

The complexity and diversity of glycosylation significantly complicates the linkage between genetic sequence and mature, active proteins. Glycobiology-focused proteomics, or glycomics, requires the development of novel approaches and tools directed at the special challenges of glycobiology. Among post-translational modifications, glycosylation is the only one that requires structural characterization of the modifying moiety beyond noting its presence. Strategies for separation, profiling, quantitation, and detailed characterization of carbohydrate structures are central challenges. Informatics tools are needed for data handling and reduction, correlation of carbohydrate and protein information, and a variety of other purposes. Discovery-based analytical tools that can survey the complexities of glycosylation on a system-wide basis may have significant biological impact.

The goals of this PA are deliberately discussed with respect to fundamental challenges, rather than in relation to specific technologies, in order to emphasize the overriding importance of surmounting obstacles, irrespective of the analytical strategy adopted to pursue those solutions. This solicitation is open to unconventional or alternative approaches.

This PA uses the SBIR and STTR mechanisms, which are set-aside programs. As an applicant, you will be solely responsible for planning, directing, and executing the proposed project. Future unsolicited, competing- continuation applications based on this project will compete with all SBIR/STTR applications and will be reviewed according to the customary peer review procedures.

This PA uses just-in-time concepts. It also uses the modular budgeting format. Specifically, if you are submitting an application budget of $100,000 total costs (direct, F&A and fee) or less, use the modular format and instructions as described in the current SBIR/STTR Omnibus Solicitation. Otherwise follow the instructions for non-modular budget research grant applications. This program does not require cost sharing as defined in the current NIH Grants Policy Statement at http://grants.nih.gov/grants/policy/nihgps_2003/NIHGPS_Part2.htm#matching_or_cost_sharing.

Applications may be submitted for support as Phase I STTR (R41) or Phase I SBIR (R43) grants; Phase II STTR (R42) or Phase II SBIR (R44) grants; or the SBIR/STTR FAST-TRACK option as described in the SBIR/STTR Omnibus Solicitation. Phase II applications in response to this PA will only be accepted as competing continuations of previously funded NIH Phase I SBIR/STTR awards. The Phase II application must be a logical extension of the Phase I research but not necessarily a Phase I project supported in response to this PA.

The PHS 398 research grant application must be used for all SBIR/STTR Phase I, Phase II and Fast-Track applications (new and revised.) Effective October 1, 2003, applications must have a DUN and Bradstreet (D&B) Data Universal Numbering System (DUNS) number as the Universal Identifier when applying for federal grants or cooperative agreements. The DUNS number can be obtained by calling 866-705-5711 or through the website at http://www.dunandbradstreet.com/. The DUNS number should be entered on line 11 of the face page of the PHS 398 form. The PHS 398 is available at http://grants.nih.gov/grants/funding/phs398/phs398.html. Prepare your application in accordance with the SBIR/STTR Omnibus Solicitation and the PHS 398. Helpful information for advice and preparation of the application can be obtained at: http://grants.nih.gov/grants/funding/sbir-grantsmanship.pdf. The NIH will return applications that are not submitted on the 5/2001 version of the PHS 398. For further assistance contact GrantsInfo, 301-435-0714, e-mail: GrantsInfo@nih.gov. The title and number of this PA must be typed on line 2 of the face page of the application.

The CSR will not accept any application in response to this PA that is essentially the same as one currently pending initial review unless the applicant withdraws the pending application. The CSR will not accept any application that is essentially the same as one already reviewed. This does not preclude the submission of a substantial revision of an unfunded version of an application already reviewed, but such application must include an Introduction addressing the previous critique.

Receipt and review schedule: see http://grants.nih.gov/grants/funding/sbirsttr_receipt_dates.htm.

Contact: Douglas M. Sheeley, Division of Biomedical Technology, National Center for Research Resources, 6701 Democracy Blvd, MSC 4874, Bethesda, MD 20892-4874 USA, 301-435-0755, fax: 301-480-3659, e-mail: sheeleyd@mail.nih.gov; Pamela A. Marino, NIGMS, Rm 2As.43k, Natcher Building, Bethesda, MD 20892-6200 USA, 301-594-3827, fax: 301-480-2802, e-mail: marinop@nigms.nih.gov; Susan E. Old, Division of Heart and Vascular Disease, NHLBI, 6701 Rockledge Dr, MSC 7940, Bethesda, MD 20892-7940 USA, 301-435-1802, fax: 301 480-1335, e-mail: olds@nhlbi.nih.gov; Danilo A. Tagle, Neuroscience Center, NINDS, Rm 2133, 6001 Executive Blvd, Bethesda, MD 20892-0001 USA, 301-496-5745, fax: 301-402-1501, e-mail: tagled@ninds.nih.gov.

Reference: PA No. PA-04-089

## Intellectual Property Rights in Genetics and Genomics

The purpose of this RFA is to encourage the study of the role of laws and policies regarding intellectual property rights in genetics and genomics research and development, and the effect of such laws and policies on progress in these fields and on commercialization, drug development, health care delivery, and the public health.

Since its inception, the Human Genome Project has attempted to follow a policy of free and open access to genetic and genomic data e.g., National Human Genome Research Institute (NHGRI) Policy Regarding Intellectual Property of Human Genomic Sequence (April 9, 1996), http://www.genome.gov/10000926; NHGRI Policy on Human Genomic Sequence Data (Dec. 21, 2000), http://www.genome.gov/10000910. The National Institutes of Health (NIH) policy recognizes the appropriateness of intellectual property protections for discoveries that are associated with useful products, but promotes the free dissemination of research tools whenever possible, especially when the prospect of commercial gain is remote (Report of the National Institutes of Health (NIH) Working Group on Research Tools, http://www.nih.gov/news/researchtools/).

Over the past three decades, however, many patents have been granted on gene sequences and other types of basic information derived from genetic sequence. For some, this has generated apprehension that gene patents are being granted too broadly or freely, especially for foundational tools. The concern is that the too-liberal issuance of such patent rights, especially when coupled with exclusive licensing practices, will result in the imposition of reach-through restrictions or excessive fees, and inhibit investigators from conducting additional research with these tools. This, it is feared, will ultimately be to the detriment of advances in medical research and to public health.

In January 2001, partly in response to a letter from the NIH urging the implementation of stricter criteria for the issuance of biotechnology patents, the U.S. Patent and Trademark Office revised its guidelines to patent examiners regarding patents on DNA sequence and sequence-derived intellectual property, effectively “raising the bar” on utility standards in this area [U.S. Patent and Trademark Office, Utility Examination Guidelines, Fed Reg 66(4) (January 5, 2001)]. However, questions remain about whether this revision raised the “bar” high enough to serve the public interest. An example of the potential problem is the recent acquisition and aggressive pursuit by Genetic Technologies Limited (GTG), an Australian company, of exceptionally broad global patent protection covering the use of information to derive risks of disease in all non-coding regions of the genome [see Nature (2003) 423:105]. While this is perhaps an extreme example (and the validity of GTG’s patents has not yet been tested in the courts), other controversial cases can also be cited (e.g., the Myriad Genetics BRCA1 patent, the University of Miami Canavan disease patent, the CCR5 HIV co-receptor gene patent). Such cases are increasingly leading genetics and genomics researchers, business entities, health care providers, and consumers to question how the balance between providing intellectual property protection and fostering biomedical innovation can best be attained.

Issues regarding the appropriate scope of protection for intellectual property rights in genetics and genomics research and development will only increase in complexity as progress in these fields continues. For example, large-scale proteomics efforts [such as protein biomarker discovery projects, the NIGMS Protein Structure Initiative (http://www.nigms.nih.gov/psi/) and initiatives to characterize protein-protein interactions] will generate new types of potentially patentable information, and with this information, new intellectual property challenges. Such challenges will also arise in several areas of research being emphasized under the new NIH Roadmap Initiative (http://nihroadmap.nih.gov/). For example, in the “chemical genomics” area, questions will arise about whether patents should be filed on the compounds that will be discovered or whether to place such compounds in the public domain, and about how pricing should be determined should a compound discovered through this process end up as a drug. In the bioinformatics and computational biology area, questions will arise about how best to promote the widespread distribution of new software to be developed (e.g., using an open source model of licensing or some other model).

Anticipating the growing need to confront questions of this type, the NHGRI has identified addressing intellectual property issues as one of the “Grand Challenges” for the future of genomics. Specifically, the Institute’s document “A Vision for the Future of Genomics Research,” [Nature (2003) 422:835–847], also available at: http://www.genome.gov/11006873), called for “the development of policy options in the area of intellectual property that will facilitate the widespread use of genetic and genomic information in both research and clinical settings.” To be maximally informed and effective, however, the development of such policy options must be based on a solid and broad-based body of theoretic and empiric data. While a number of studies already conducted or now underway provide a good preliminary foundation on which to build, there is a clear need for additional research and scholarship in this area.

In 2004, the Board on Science Technology and Economic Policy (STEP Board) and the Science, Technology, and Law Program of the National Academies of Sciences convened a committee on Intellectual Property in Genomic and Protein Research and Innovation (the “NAS Committee”). The NAS Committee’s charge is to review the patenting and licensing of human genetic material and proteins and their implications for biomedical research, therapeutic and diagnostic products, and medical practice. The NAS Committee is expected to release its report in the Summer of 2005, but there will clearly be a need for other, more in depth, examinations and analyses of these issues, by investigators from a broad range of disciplines.

To assist in addressing this need, the NHGRI proposes a new initiative to encourage the study of the role of laws and policies regarding intellectual property rights in genetics and genomics research and development, and the effect of such laws and policies on progress in these fields and on commercialization, drug development, health care delivery, and public health. The initiative is designed to support rigorous, carefully focused legal, statistical, economic, political science, historical, and other social scientific investigations, both theoretical and empirical.

As used in this RFA, the term “genetics and genomics” includes genomics (broadly defined to include both nucleic acid and protein products of large-scale analyses of the human and other genomes and methods for identifying and analyzing them) and human molecular genetics. The term is not, however, meant to include all of biotechnology, although the line between genomics and biotechnology is frequently hard to define. For example, the term “genetics and genomics subject matter” includes the following: (1) Both individual elements of data and comprehensive databases or other resources regarding genes and gene fragments; gene regulatory sequences; ESTs; SNPs; haplotypes; proteins and protein structures; protein-protein interactions; cellular pathways; computational models of the cell; gene expression profiling (microarrays); small molecules; and mouse (or other animal) knockouts. (2) The relationships between diseases or traits and genes, SNPs, haplotypes, or proteins; the relationships among genotype, environment, and phenotype (e.g., in large databases); and the use of such information in diagnostics. (3) Fundamental tools or methods for the production or analysis of data or databases of the types listed above, the bioinformatics software to probe the databases, and the algorithms that the software elaborates. The term “genetics and genomics subject matter” as used in this initiative does not, however, include such subject matter as biomedical devices, engineered tissues, stem cells, large-scale cell culture, whole organism cloning, or individual treatment applications.

Some examples of appropriate topic areas, with examples of specific research questions for each area, are listed below. Investigators are welcome to propose research in one or more of these topic areas, or in similar areas. Investigators should not be constrained by the specific research questions included on this list. The focus of the research, however, should remain on intellectual property rights to genetics and genomics-related subject matter, and should not be so broad as to encompass other major areas of biotechnology. (1) Types of Intellectual Property Rights and Related Policy Implications. What types of intellectual property rights to genetics and genomics-related subject matter are being, or should be, sought, obtained, or refused? What types of entities are seeking, obtaining, or being refused, intellectual property rights in this field? What are, or should be, the standards for novelty, non-obviousness, and utility in this field? What is, or should be, the breadth of the claims in this field? Do intellectual property rights to genetics and genomics-related subject matter benefit the public when there is no identifiable product? What has been the effect of intellectual property rights in this field on research in the private sector? What are the mechanisms, existing or proposed as well as legal or business custom, for protecting information contained in databases generally, and what are the policy implications of allowing or refusing protection for genomic and genetic databases, whether through intellectual property or sui generis protection? What is, or should be, the role of patents, copyrights, trade secrets, and sui generis intellectual property rights for various data types? How do the laws governing patents, copyrights, trade secrets, and sui generis intellectual property rights act as an incentive, a disincentive, or a neutral factor in determining the planning, content, and progress of genetics and genomics research and development programs? (2) Ownership and Assignment of Intellectual Property Rights and Related Policy Implications. What are, or should be, the mechanisms for exploiting intellectual property rights to genetics and genomics-related subject matter? How frequently are, or should, such rights be assigned (e.g., sold, or licensed exclusively or non-exclusively to third parties)? What are, or should be, the usual mechanisms of such assignments? Who are, or should be, the usual parties to such assignments? To what extent would genetics and genomics subject matter be treated differently if the corresponding intellectual property rights were not assigned? What are, or should be, the practices of biotechnology and pharmaceutical companies regarding the sharing of commercially valuable data? What are, or should be, the practices of universities regarding the sharing of commercially valuable data (government funded and non-government funded)? How have universities interpreted the Bayh-Dole Act, and what has been the impact of Bayh-Dole on genetics and genomics research? Are, or should, assignments in this field under Bayh-Dole typically be pursuant to employment contract or policies, or the result of arms-length negotiations? What is the practical impact of restrictions or limitations on the ownership of intellectual property rights imposed by government funding agencies (such as “Declaration of Exceptional Circumstances”)? How will the mechanisms of assignment of intellectual property rights, and restrictions on such assignment, likely affect genetics and genomics subject matter in the future? (3) Licensing Practices and Related Policy Implications. What are the categories of genetics and genomics subject matter for which intellectual property rights are licensed or may be licensed in the future? What are the relative numbers of intellectual property rights involving genetics and genomics subject matter that are subject to licensing arrangements? What are the terms of such licenses (including exclusivity versus non-exclusivity, royalty rates, fields of use restrictions, etc.), and who are the parties to such agreements? What are the structures for such licensing arrangements (e.g., cross-licensing, block or blanket licenses, compulsory licenses, etc.)? What are the structures and operation of patent pools? How are end user license agreements (EULAs) attached to the sale of research tools being used, and how broad are their “reach-through” provisions? To what extent might the genetics and genomics subject matter be differently treated if the corresponding intellectual property rights were not licensed or were not disclosed and treated as a trade secret? How are the planning, content, and progress of genetics and genomics research and development programs affected by refusals to license or offers to license on unacceptable terms? How does the way in which genetics and genomics subject matter is licensed affect the prospects for commercialization? What is the effect of being required to obtain multiple licenses to conduct some types of research or clinical tests? Does an open source model of licensing genomic software tools increase the usefulness of the tools and improve their acceptance in the research community? Are intellectual property rights involving genetics and genomics subject matter to which licensing arrangements pertain more or less likely to be involved in infringement litigation? What would be the policy implications of limiting exclusive licenses in the field of genetics and genomics to therapeutics and vaccines (i.e., excluding diagnostics)? (4) Enforcement and Related Policy Implications. What are the categories of genetics and genomics subject matter for which the intellectual property rights have been involved in administrative or judicial action? What legal issues have been raised in such lawsuits, and who have been the parties to such lawsuits? What has been the resolution of such cases (e.g., dismissal, settlement, administrative action, trial verdict or judgment, appellate judgment, remedies and relief awarded, etc.)? What are the relative numbers of intellectual property rights involving genetics and genomics subject matter that have been filed in various forums? What are the numbers of intellectual property rights involving genetics and genomics subject matter that have been challenged but that do not actually reach litigation? How frequently are cease and desist letters issued, and how do universities or companies respond to them? How have the planning, content, and progress of genetics and genomics research and development programs been affected by threat, actual or perceived, of infringement litigation? What strategies are employed to allocate the risk of, to prepare for, or to defend against, infringement litigation? What impact has Madey v. Duke, 64 USPQ2d 1737, 307 F.3d 1351 (Fed Cir 2002), cert. Denied, 156 L.3d. 656 (2003), interpreting the experimental use (research) exemption to patent infringement in the context, had in the context of academic research? What would be the policy implications of formalizing a research exemption in the patent law? (5) International Issues and Related Policy Implications. What are the categories of genetics and genomics subject matter for which intellectual property rights have been or may be sought both in the United States and abroad? How does the operation of intellectual property rights involving genetics and genomics subject matter differ in the United States from other countries (e.g., what are the differences in the criteria for patentability applied in the U.S. and by other major patent offices, such as in Europe and Japan)? What mechanisms of procurement, ownership, licensing, and enforcement (or restrictions on these activities) exist only in other countries, and what are the advantages and disadvantages of such? How are international treaty obligations likely to affect the laws and customs in the United States governing intellectual property rights to genetics and genomics related subject matter? How do territorial and jurisdictional limitations on intellectual property rights affect the planning, content, and progress of genetics and genomics research and development programs? (6) Overarching Issues. Has the planning, content, and progress of genetics and genomics research and development programs been enhanced, or conversely chilled, by intellectual property rights? Have intellectual property rights positively or negatively affected the quantity and quality of the publication of scientific advances involving genetics and genomics, or the timing of data release and publication? What are the legal and practical implications for unfettered research activities (e.g., the significance of a bona fide research use exemption to patent infringement, a fair use defense to copyright infringement, a reverse engineering exception to trade secret misappropriation, etc.)? Are existing mechanisms of protection of intellectual property rights to genetics and genomics related subject matter adequate or inadequate to the task of striking the proper balance between intellectual property rights and open access to devices, methods, products and data involved in genetics and genomics research and development? How have intellectual property rights to genetics and genomics-related subject matter positively or negatively affected public access to health care (e.g., accelerated or delayed the commercial availability of diagnostics or treatments, increased or decreased their cost, etc.)?

A major goal of this initiative is to help expand the research base necessary to inform the future development of policy options regarding intellectual property in the contexts of genetics and genomics research and development. In this sense, the proposed development of policy options by applicants to this initiative is not required, but is encouraged when feasible. Investigators may propose to examine existing databases related to biotechnology and intellectual property rights or to gather new empirical data. However, proposals that are primarily dependent on data mining efforts should identify and incorporate innovative analytical methodologies to interpret the data.

Although applications for proposals to examine issues regarding intellectual property, genetics, and genomics in the specific context of differing cultures and belief systems are beyond the scope of this initiative, the NHGRI encourages research on these topics as part of its regular research program in the area of Ethical, Legal, and Social Implications (ELSI). Applicants interested in conducting research on such topics are strongly encouraged to consider submitting R01 or R03 applications under one of the appropriate standing NHGRI PAs for the ELSI Program. See http://grants.nih.gov/grants/guide/pa-files/PA-04-050.html (R01 Program Announcement); http://grants.nih.gov/grants/guide/pa-files/PA-04-051.html (R03 Program Announcement).

This RFA will use NIH R01 and R03 award mechanisms. Applicants are solely responsible for planning, directing, and executing the proposed project. This RFA is a one-time solicitation. Future unsolicited, competing-continuation applications based on this project will compete with all investigator-initiated applications and will be reviewed according to the customary peer review procedures. The earliest anticipated award date is 15 July 2005. Applications that are not funded in the competition described in this RFA may be resubmitted as new investigator-initiated applications using the standard receipt dates for new applications described in the instructions to the PHS 398 application.

This RFA uses just-in-time concepts. It also uses the modular as well as the non-modular budgeting formats (see http://grants.nih.gov/grants/funding/modular/modular.htm).

Annual meetings of investigators will be held. This will facilitate the sharing of information, encourage collaboration, reduce possible duplication of effort, and promote more rapid dissemination of research findings. The initial meeting will take place shortly after the awards are made. Funds for travel to these meetings for up to two investigators per year should be included in the requested budget.

Prospective applicants are asked to submit a letter of intent that includes the following information: descriptive title of the proposed research; name, address, and telephone number of the Principal Investigator; names of other key personnel; participating institutions; number and title of this RFA. Although a letter of intent is not required, is not binding, and does not enter into the review of a subsequent application, the information that it contains allows Institute Center (IC) staff to estimate the potential review workload and plan the review.

Applications must be prepared using the PHS 398 research grant application instructions and forms (rev. 5/2001). Applications must have a DUN and Bradstreet (D&B) Data Universal Numbering System (DUNS) number as the Universal Identifier when applying for federal grants or cooperative agreements. The DUNS number can be obtained by calling 866-705-5711 or through the website at http://www.dunandbradstreet.com/. The DUNS number should be entered on line 11 of the face page of the PHS 398 form. The PHS 398 document is available at http://grants.nih.gov/grants/funding/phs398/phs398.html in an interactive format. For further assistance contact GrantsInfo, 301-435-0714, e-mail: GrantsInfo@nih.gov.

The RFA label available in the PHS 398 (rev. 5/2001) application form must be affixed to the bottom of the face page of the application. Type the RFA number on the label. Failure to use this label could result in delayed processing of the application such that it may not reach the review committee in time for review. In addition, the RFA title and number must be typed on line 2 of the face page of the application form and the YES box must be marked. The RFA label is also available at: http://grants.nih.gov/grants/funding/phs398/label-bk.pdf.

The Center for Scientific Review (CSR) will not accept any application in response to this RFA that is essentially the same as one currently pending initial review, unless the applicant withdraws the pending application. However, when a previously unfunded application, originally submitted as an investigator-initiated application, is to be submitted in response to an RFA, it is to be prepared as a new application. That is, the application for the RFA must not include an introduction describing the changes and improvements made, and the text must not be marked to indicate the changes from the previous unfunded version of the application.

Letters of intent are due 21 October 2004, with applications due 18 November 2004. The earliest anticipated start date is 15 July 2005.

Contact: Jean E. McEwen, NHGRI, Division of Extramural Research, Ethical, Legal, and Social Implications Program, 5635 Fishers Lane, Suite 4076, MSC 9305, Bethesda, MD 20892-9305 USA, until 28 June 2004: 301-402-4997, after 28 June 2004: 301-496-7531, fax: 301-402-1950, e-mail: jm522n@nih.gov; Rudy O. Pozzatti, NHGRI, Scientific Review Branch, 5635 Fishers Lane, Suite 4076, MSC 9306, Bethesda, MD 20892-9306 USA, 301-402-0838, fax: 301-435-1580, e-mail: rp7s@nih.gov.

Reference: RFA No. RFA-HG-04-004

## SBIR/STTR: Circulating Cells and DNA in Cancer Detection

Notice: This Request for Application (RFA) must be read in conjunction with the current Omnibus Solicitation of the National Institutes of Health (NIH), Centers for Disease Control and Prevention (CDC), and Food and Drug Administration (FDA) for Small Business Innovation Research (SBIR) Small Business Technology Trandfer (STTR) Grant Applications. The solicitation (see http://grants.nih.gov/grants/funding/sbirsttr1/index.pdf or http://grants.nih.gov/grants/funding/sbirsttr1/index.doc) contains information about the SBIR and STTR programs, regulations governing the programs, and instructional information for submission. All of the instructions within the SBIR/STTR Omnibus Solicitation apply with the exception of the following: special receipt dates, and initial review convened by the National Cancer Institute (NCI) Division of Extramural Activities.

The Division of Cancer Prevention of the NCI invites small business applications for research projects to develop novel technologies for capturing, enriching, and preserving exfoliated abnormal cells and circulating DNA from body fluids or effusions and to develop methods to concentrate these cells and DNA for cancer biomarker detection.

In body fluids, such as sputum, the number of exfoliated tumor cells is often low compared to the number of normal cells, making it difficult to detect these abnormal cells by routine cytopathology. Separation of dysplastic cells from degenerating cells and cells undergoing non-specific reactive changes is problematic. Moreover, exfoliated cells are frequently contaminated with normal cells, bacteria, and cellular debris. Therefore, enrichment methods are needed to allow for routine detection and molecular analysis of small numbers of exfoliated cells.

Circulating extracellular DNA was first reported in 1948. It has been shown that the circulating DNA in the blood of cancer patients has genetic characteristics identical to those of the primary tumors. Thus, circulating DNA is an important material that may be useful for cancer detection. Currently available methods for isolating undegraded circulating DNA are limited, and there is a need to develop novel methods which improve the yield of undegraded DNA and to adapt detection assays so that this DNA can be used to detect mutations, microsatellite instabilities, loss of heterozygosity, epigenetic changes, and other molecular genetic changes.

This RFA will utilize the SBIR and STTR mechanisms, but will be run in parallel with a program announcement of identical scientific scope (PA-04-035) that will utilize the exploratory/developmental (R21) grant mechanism.

Cellular and molecular changes that ensue during tumor progression occur over a number of years and in an apparently stochastic manner. For example, it takes an average of 15 to 20 years for a small adenomatous polyp to become malignant. Prior to the appearance of a morphologically identified precancerous lesion, numerous genetic and molecular alterations have occurred. During the early stages of cancer development, there is a window of opportunity to detect precancerous cells with genetic or molecular biomarkers that identify and characterize their progression towards cancer. Finding molecular and genetic biomarkers of malignancy is an extraordinary opportunity for the NCI and is particularly important in detecting the emergence of precancerous cell populations. In these earliest stages of neoplasia, lesions are more likely to be amenable to eradication. This principle has been well-demonstrated in cervical neoplasia, where screening for dysplastic exfoliated cells can result in a 70 percent or greater reduction in mortality due to cervical cancer. Detection of genetic abnormalities in preneoplastic lesions poses challenges because of the small size of lesions, the heterogeneity of precancerous cells, and the relatively low number of abnormal cells compared to normal cells.

More than 80 percent of human tumors (e.g. colon, lung, prostate, oral cavity, esophagus, stomach, uterine cervix, and bladder) originate from epithelial cells, often at a mucosal surface, and are clonal in origin.Cells from these tumors exfoliate spontaneously into blood, sputum, urine, and various effusions. Abnormalities within these exfoliated cells could be used to detect and identify precancerous lesions or very early stage cancers if highly sensitive technologies were available to identify the presence of a few abnormal cells among millions of normal cells. For example, PCR has been used to detect mutant DNAs in neoplastic exfoliated cells; mutations have been detected in ras genes present in stool samples obtained from patients with colorectal cancer, and in p53 from the urine of patients with bladder cancer and in the sputa of patients with lung cancer. Assays to detect genetic mutations, microsatellite instability, or hypermethylation may be adapted for use with exfoliated cells. As these assays are complex and technically challenging, their general use will require the development of novel technologies for isolating and enriching abnormal exfoliated cells.

Studies performed in the early 1970s showed that increased quantities of DNA are found in the plasma of patients suffering from different malignancies, but it was not until the 1990s that this circulating DNA was shown to exhibit tumor-related alterations. Mutant DNA has been found in the plasma of patients with colorectal, pancreatic, biliary tree, skin, head-and-neck, lung, breast, kidney, ovarian, nasopharyngeal, liver, bladder, gastric, prostate, and cervical cancers as well as in haematologic malignancies. Allelic imbalance (AI), which involves the loss or gain of chromosomal regions, is found in many cancers. AI can be detected in genomic tumor DNA released into the blood after cellular necrosis or apoptosis. These observations indicate that plasma/serum may be a suitable specimen source for noninvasive diagnostic, prognostic, and follow-up tests for cancer.

Precancerous exfoliated cells can be identified by cytologic examination of washings or brushings from bronchi, oral cavity, esophagus, stomach, bile and pancreatic ducts, as well as of sputum and urine specimens. However, the detection of these exfoliated cancer cells by routine cytopathological examination is very difficult because the number of abnormal cells in the specimens is usually very low compared to the number of normal cells. It is also difficult to distinguish low grade dysplasia from non-specific reactive or inflammatory changes due to the low sensitivity and specificity of current diagnostic methodologies. This is particularly true of urine cytology, where most low-grade papillary lesions are missed by cytologic examination. Even with new PCR-based technologies with enhanced sensitivity, current technologies for isolating exfoliated cells are too inefficient to be of practical utility. Therefore, the development of novel, high-throughput, sensitive technologies for sample preparations is a prerequisite for the successful detection of the small number of exfoliated cells or of the small amounts of DNA, RNA and proteins in these cells.

There are a variety of approaches to detect and analyze precancerous and cancerous cells in body fluids [e.g., cytopathological analysis, morphometric analysis, molecular biomarkers for specific receptors or genetic changes, Fluorescence in Situ Hybridization (FISH) analysis, or PCR-based analysis].The selection of approach, in many instances, depends on the type of biological specimens (sputum, bronchial washing, cervical brushing, voided urine, etc.). Given that the concentration of the atypical epithelial cells can be very low compared to that of normal cells, all of these approaches require between 1 to 10,000 and 1 million enrichments of the atypical cells. Currently, there are two broad categories of enrichment methods: mechanical (centrifugation, cytospin, sucrose gradients, etc.) and antibody-based selection with mechanical separation (FACS – flow-assisted cell sorting, MACS - magnetic assisted cell sorting, etc.). While these two types of enrichment processes can be used in series to improve the yield, none of the currently available methods achieve sufficient enrichment of atypical cells to allow them to be routinely used for cancer detection.

The single largest barrier to using circulating DNA for cancer detection is the amount of circulating undegraded DNA that can be isolated is low, making it unsuitable for currently available assay technologies. Several factors affect the yield and purity of circulating DNA. Intracellular nuclease activity in both apoptotic and necrotic cells in a particular organ affect the degree of DNA degradation found in body fluids. Also, the degree to which a particular tissue is represented in the total circulating DNA is dependent on the mechanism and efficiency by which apoptotic cells are eliminated from the tissue.

As with any other diagnostic technique, practical application of circulating DNA technology is dependent on concurrent increase in the sensitivity and reproducibility of molecular based-assays. The potential use of circulating DNA for cancer detection could be greatly enhanced by developing isolation methods that result in less degradation and by adapting assay methods to use the low amounts that can be isolated. Because of the limitations of “conventional” markers, there has been a search for additional sources of specificity so as to expand the target pool of cancer-associated molecules. Circulating cells and DNA offer such opportunity for detection molecular aberrations in plasma/serum, or other body fluids, that accurately reflect the situation in primary tumor. This will, however, require the development of methodological consistencies so as to allow valid comparisons between various assays based on circulating cells or DNA.

The primary purpose of this initiative is to encourage the development of technologies for isolating and characterizing exfoliated cells, circulating cells, and plasma/serum DNA. A secondary purpose is the analytical validation of existing and/or newly developed technologies for their usefulness in cancer detection. Analytical validation refers to the measurement of sensitivity and reproducibility of the proposed assay/technology. The long-term goal of the technology development is to identify a panel of well-characterized biomarkers derived from exfoliated cells and/or circulating DNA that can be sampled in a clinical setting. These methodologies will be tested and validated in future population-based clinical trials, and integrated into a comprehensive information system that will be developed under the Early Detection Research Network (www.cancer.gov/edrn). In pursuit of these goals, the NCI invites applications which address the following areas: 1) Development of high-throughput, high-yield technologies for isolating exfoliated cells, circulating cells and DNA in body fluids; 2) Development of methods for enrichment and preservation of exfoliated cells, circulating cells and DNA isolated from body fluids; 3) Development of sensitive, high-throughput molecular, cytomorphometric, immunologic, and other relevant technologies to isolate and characterize tumor cells in malignant effusions for detection of low tumor burden, to help distinguish reactive cells from tumor cells, and to perform accurate assays on circulating DNA; 4) Validation of the sensitivity and reproducibility of current technologies for isolating and characterizing exfoliated cells, circulating cells and DNA isolated from body fluids.

This RFA uses the SBIR and STTR mechanisms, which are set-aside programs. As an applicant, you will be solely responsible for planning, directing, and executing the proposed project. Future unsolicited, competing-continuation applications based on this project will compete with all SBIR/STTR applications and will be reviewed according to the customary peer review procedures. The anticipated award date is approximately 9–11 months from the respective receipt date. Applications that are not funded in the competition described in this RFA may be resubmitted as new SBIR/STTR applications using the standard receipt dates for new applications described in the current SBIR/STTR Omnibus Solicitation. As there are multiple receipt dates, it is possible that an unfunded application can be resubmitted under this RFA as a revised application.

This RFA uses just-in-time concepts. It also uses the modular budgeting as well as the non-modular budgeting formats. Specifically, if you are submitting an application budget of $100,000 total costs (direct, F&A and fee) or less, use the modular budget format. For applications requesting more than $100,000, use the non-modular budget format. Instructions for both are described in the current SBIR/STTR Omnibus Solicitation. This program does not require cost sharing as defined in the current NIH Grants Policy Statement at http://grants.nih.gov/grants/policy/nihgps_2003/NIHGPS_Part2.htm.

Except as otherwise stated in this RFA, awards will be administered under NIH grants policy as stated in the NIH Grants Policy Statement, December 2003, available at http://grants.nih.gov/grants/policy/nihgps_2003/.

Applications may be submitted for support as Phase I STTR (R41) or Phase I SBIR (R43) grants; Phase II STTR (R42) or Phase II SBIR (R44) grants; or the SBIR/STTR FAST-TRACK option as described in the SBIR/STTR Omnibus Solicitation. Phase II applications in response to this RFA will only be accepted as competing continuations of previously funded NIH Phase I SBIR/STTR awards. A Phase II application must be a logical extension of the Phase I research but not necessarily a Phase I project supported in response to this RFA. Fast Track applications will benefit from expedited evaluation of progress following the Phase I feasibility study for transition to Phase II funding for expanded developmental work.

Prospective applicants are asked to submit a letter of intent that includes the following information: descriptive title of the proposed research; name, address, and telephone number of the Principal Investigator; names of other key personnel; participating institutions, number and title of this RFA. Although a letter of intent is not required, is not binding, and does not enter into the review of a subsequent application, the information that it contains allows IC staff to estimate the potential review workload and plan the review.

The PHS 398 research grant application must be used for all SBIR/STTR Phase I, Phase II, and Fast-Track applications (new and revised). Effective 1 October 2003, applications must have a Dun and Bradstreet (D&B) Data Universal Numbering System (DUNS) number as the Universal Identifier when applying for federal grants or cooperative agreements. The DUNS number can be obtained by calling 866-705-5711 or through the website at http://www.dunandbradstreet.com/. The DUNS number should be entered on line 11 of the face page of the PHS 398 form. The PHS 398 is available at http://grants.nih.gov/grants/funding/phs398/phs398.html. Prepare your application in accordance with the SBIR/STTR Omnibus Solicitation and the PHS 398. Helpful information for advice and preparation of the application can be obtained at http://grants.nih.gov/grants/funding/sbirgrantsmanship.pdf. The NIH will return applications that are not submitted on the 5/2001 version of the PHS 398. For further assistance, contact GrantsInfo 301-435-0714; e-mail: GrantsInfo@nih.gov.

Applications hand delivered by individuals to the NCI will no longer be accepted. This policy does not apply to courier deliveries (i.e., FEDEX, UPS, DHL, etc.) (see http://grants.nih.gov/grants/guide/notice-files/NOT-CA-02-002.html). This policy is similar to and consistent with the policy for applications addressed to Centers for Scientific Review as published in the NIH Guide Notice at http://grants.nih.gov/grants/guide/notice-files/NOT-OD-02-012.html.

The Center for Scientific Research (CSR) will not accept any application in response to this RFA that is essentially the same as one currently pending initial review unless the applicant withdraws the pending application. The CSR will not accept any application that is essentially the same as one already reviewed. However, when a previously unfunded application, originally submitted as an investigator-initiated application, is to be submitted in response to an RFA, it is to be prepared as a new application. That is, the application for the RFA must not include an introduction describing the changes and improvements made, and the text must not be marked to indicate the changes from the previous unfunded version of the application.

Letters of Intent are due 17 January 2005, 16 May 2005, and 14 September 2005. Applications are due 14 February 2005, 13 June 2005, and 12 October 2005. The earliest anticipated start dates are January 2006, April 2006, and July 2006.

Contact: Sudhir Srivastava, Division of Cancer Prevention, NCI, 6130 Executive Blvd, EPN Rm 3144, Bethesda, MD 20892-0001 USA, Rockville, MD 20852 (for express/courier service), 301-496-3983, fax: 301-402-8990, e-mail: ss1a@nih.gov; (for peer review issues) Referral Officer, NCI, Division of Extramural Activities, 6116 Executive Blvd, Rm 8041, MSC 8329, Bethesda, MD 20892-8329 USA, Rockville, MD 20852 (for express/courier service), 301-496-3428, fax: 301-402-0275, e-mail: ncirefof@dea.nci.nih.gov.

Reference: RFA No. RFA-CA-06-001

